# PAPP-A2 deficiency does not exacerbate the phenotype of a mouse model of intrauterine growth restriction

**DOI:** 10.1186/s12958-018-0376-4

**Published:** 2018-06-12

**Authors:** Julian K. Christians, Kendra I. Lennie, Maria F. Huicochea Munoz, Nimrat Binning

**Affiliations:** Department of Biological Sciences, Simon Fraser University, Burnaby, BC Canada

**Keywords:** Placenta, Pregnancy, Preeclampsia, Intrauterine growth restriction, Pregnancy associated plasma protein, Matrix metalloproteinase, Insulin-like growth factor

## Abstract

**Background:**

Pregnancy-associated plasma protein-A2 (PAPP-A2) is consistently upregulated in the placentae of pregnancies complicated by preeclampsia and fetal growth restriction. The causes and significance of this upregulation remain unknown, but it has been hypothesized that it is a compensatory response to improve placental growth and development. We predicted that, if the upregulation of PAPP-A2 in pregnancy complications reflects a compensatory response, then deletion of *Pappa2* in mice would exacerbate the effects of a gene deletion previously reported to impair placental development: deficiency of matrix metalloproteinase-9 (MMP9).

**Methods:**

We crossed mice carrying deletions in *Pappa2* and *Mmp9* to produce pregnancies deficient in one, both, or neither of these genes. We measured pregnancy rates, number of conceptuses, fetal and placental growth, and the histological structure of the placenta.

**Results:**

We found no evidence of reduced fertility, increased pregnancy loss, or increased fetal demise in *Mmp9*^*−/−*^ females. In pregnancies segregating for *Mmp9*, *Mmp9*^*−/−*^ fetuses were lighter than their siblings with a functional *Mmp9* allele. However, deletion of *Pappa2* did not exacerbate or reveal any effects of *Mmp9* deficiency. We observed some effects of *Pappa2* deletion on placental structure that were independent of *Mmp9* deficiency, but no effects on fetal growth. At G16, male fetuses were heavier than female fetuses and had heavier placentae with larger junctional zones and smaller labyrinths.

**Conclusions:**

Effects of *Mmp9* deficiency were not exacerbated by the deletion of *Pappa2*. Our results do not provide evidence that upregulation of placental PAPP-A2 represents a mechanism to compensate for impaired fetal growth.

**Electronic supplementary material:**

The online version of this article (10.1186/s12958-018-0376-4) contains supplementary material, which is available to authorized users.

## Background

Intrauterine growth restriction and preeclampsia threaten the health and wellbeing of both the fetus and the mother, affecting 5–7% of pregnancies and constituting leading causes of perinatal and maternal mortality [1]. These conditions are thought to be caused, at least in part, by abnormal placental development and function [2]. There have been enormous efforts to identify the molecular mechanisms responsible for placental dysfunction in preeclampsia and intrauterine growth restriction, with numerous studies examining placental gene expression at delivery. Pregnancy-associated plasma protein-A2 (PAPP-A2) is one of the genes most consistently found to be upregulated in preeclampsia [3–7] and is also associated with fetal growth restriction [8]. Furthermore, elevated levels of PAPP-A2 in the maternal circulation in the first trimester have also been associated with preeclampsia [9, 10]. PAPP-A2 is a protease of insulin-like growth factor binding protein 5 (IGFBP-5) [11] and is thought to regulate insulin-like growth factor (IGF) availability, although it may also function through other pathways [12]. IGFs play key roles in placental development [13], and their availability is regulated by six IGF binding proteins (IGFBPs). IGFs are released primarily through cleavage of the IGFBPs by proteases [14]. PAPP-A2 deficiency would therefore be expected to reduce IGF availability, and indeed loss-of-function mutations in humans reduce stature [15] while *Pappa2* deletion in mice reduces body size [16–18] and increases IGFBP-5 levels [19].

Despite the high expression of PAPP-A2 in mouse placenta [20], *Pappa2* deletion has no effect on pregnancy outcomes, apart from slightly reduced birthweight, which may be due to deletion in the fetus itself [16, 21]. We therefore hypothesized that the upregulation of PAPP-A2 in human pregnancy complications represents a compensatory response to increase IGF signaling to promote placental growth and development [10, 22, 23]. To test this hypothesis, we deleted *Pappa2* in a mouse model of preeclampsia and intrauterine growth restriction: deficiency of matrix metalloproteinase-9 (MMP9) [24]. We selected this model since *Mmp9* deletion impairs early placental development [24], and thus reflects “canonical” preeclampsia, rather than preeclampsia of other etiologies [25]. Deficiencies in early placental development would be expected to be ameliorated by increased IGF availability and, therefore, by increased PAPP-A2 expression. We predicted that, if the elevated expression of PAPP-A2 in preeclampsia in humans reflects a compensatory response, then deletion of *Pappa2* in mice would exacerbate effects of *Mmp9* deletion, i.e., mice null for both *Pappa2* and *Mmp9* would show a more severe phenotype than mice null for *Mmp9* only. We focused on the number and size of fetuses, as well as placental histology since these were traits expected to be affected by *Mmp9* deficiency [24] and potentially ameliorated by PAPP-A2 and increased IGF availability.

## Methods

All work was carried out in accordance with the guidelines of the Canadian Council on Animal Care and approved by the SFU University Animal Care Committee (protocol 1188B). *Pappa2* deletion mice with a C57BL/6 background were generated as previously described [16, 19]. Females homozygous for *Pappa2* deletion (*Pappa2*^*−/−*^) were crossed with a male homozygous for *Mmp9* deletion (*Mmp9*^*−/−*^) obtained from the Jackson Laboratory (stock number 007084) to produce offspring heterozygous at both genes. The first generation (F1) offspring were crossed to produce an F2 population that included mice with all nine possible genotypes (three *Mmp9* genotypes x three *Pappa2* genotypes). Females and males from the F2 population were selected based on *Mmp9* and *Pappa2* genotype for breeding experiments, and were mated as described in Table 1. Rather than using only homozygous mice, we performed a variety of crosses to make use of as many mice as possible (Table 1). Furthermore, mating type 1 with *Mmp9*^*+/−*^ males was previously reported to show a reduction in litter size and placental abnormalities [24]. Beginning at approximately 8 weeks of age, females were placed with a male for one night, checked for vaginal plugs, and removed from the male whether or not a plug was observed. If no vaginal plug was observed and/or if female weight had not increased by ~ 1 g 1 week after mating, females were paired again. F2 females were collected at day 16 of gestation (G16; where the day after mating = day 0). To obtain mice for a further cohort, some females heterozygous at both genes (*Mmp9*^*+/−*^; *Pappa2*^*+/−*^) were paired with males homozygous for both deletions (*Mmp9*^*−/−*^; *Pappa2*^*−/−*^) and not collected during pregnancy. We produced a backcross (BC) population, rather than crossing heterozygotes, to increase the number of mice homozygous for *Mmp9* and/or *Pappa2* deletion. Females and males from the BC population were mated in the same manner as the F2 population, except that females were collected at day 18 of gestation (G18). Pregnancies were sampled at G18 in case effects were apparent only after G16. 63 F2 females were mated, yielding 43 G16 pregnancies (although one was mistakenly not collected during pregnancy) whereas 24 BC females were mated, yielding 20 G18 pregnancies.

**Table 1:** *Mmp9* crosses performed in experiments

Mating type	Female MMP9 genotype	Male MMP9 genotype
0	−/−	−/−
1	−/−	+/−
2	+/−	−/−
3	+/−	+/−
4	+/+	+/+

Each type of *Mmp9* cross included either no functional *Pappa2* alleles (*Pappa2*^*−/−*^ x *Pappa2*^*−/−*^) or at least one functional *Pappa2* (achieved with various combinations of female and male genotype)

At collection, females were blood sampled by cardiac puncture and the entire uterus was placed in 4% formaldehyde solution in phosphate buffered saline for 3 days before it was dissected to count and weigh individual fetuses and placentae, and to count putative fetal resorptions (green or green/brown masses). Maternal serum vascular endothelial growth factor (VEGF) was measured by enzyme-linked immunosorbent assay (R&D Systems, MMV00). Fixed placentae were stored in 70% ethanol until embedded in paraffin. A subset of placentae were selected for sectioning, attempting to include one male and one female placenta for each female, and excluding heterozygous genotypes. Because previous work reported that both embryonic and maternal *Mmp9* deficiency affect placental development [24], we selected *Mmp9*^*−/−*^ placentae from both *Mmp9*^*−/−*^ and *Mmp9*^*+/−*^ dams, as well as *Mmp9*^*+/+*^ placentae from *Mmp9*^*+/+*^ dams. For each placenta, multiple sections (6 μm) were obtained ~ 440 μm apart, up to a maximum of 10 sections per placenta. Sections were stained with haematoxylin and eosin and the areas of the labyrinth, junctional zone and decidua were measured using ImageJ 1.48v. Damaged sections, and sections close to the edge of the placenta (i.e., where the labyrinth was mostly surrounded by junctional zone) were excluded, yielding 454 sections from 62 placentae (average: 7.3 sections per placenta). To obtain a single value for each of the labyrinth, junctional zone and decidua for each placenta, we analysed the areas from all 454 sections using a general linear model (proc GLM, SAS, Version 9.4) including terms for placenta identity and section location (i.e., close to the centre vs. further from the centre; sections further from the centre had smaller areas). From this analysis, we obtained the least squares mean for each placenta for each of the labyrinth, junctional zone and decidua.

To obtain tissue for genotyping, mice were ear-clipped at weaning or a small section of fetal tail was collected. *Pappa2* genotype [19] and fetal sex [26] were determined as previously described. *Mmp9* genotype was determined by PCR as recommended by the Jackson Laboratory (primers used: 5’-CTGAATGAACTGCAGGACGA-3′; 5’-ATACTTTCTCGGCAGGAGCA-3′; 5’-GTGGGACCATCATAACATCACA-3′; 5’-CTCGCGGCAAGTCTTCAGAGTA-3′;).

All statistical analyses were performed using general linear models (proc GLM) or repeated measures analyses (proc MIXED) in SAS, Version 9.4 (SAS Institute Inc., Cary, NC). Repeated measures analyses (with dam as a random factor) were used for placental and fetal traits where there were multiple offspring per dam, since the dam was the unit of replication.

## Results

The genotype ratios and postnatal growth of the F2 and BC populations are presented in the Additional file 1. Combining F2 and BC females, we found no evidence of reduced fertility or increased pregnancy loss in *Mmp9*^*−/−*^ females. The proportion of females that became pregnant did not differ between *Mmp9*^*−/−*^ females and other females, whether all females were analysed together (Fisher’s Exact Test *P* = 0.47) or separately based on whether at least one wild-type *Pappa2* allele was present (Fisher’s Exact Test *P* = 1.00) or not (Fisher’s Exact Test *P* = 0.27) (Table 2). Similarly, the proportion of females that had at least one failed mating (i.e., a vaginal plug was detected following mating, but pregnancy did not develop) did not differ between *Mmp9*^*−/−*^ females and other females. This was true whether all females were analysed together (Fisher’s Exact Test *P* = 0.47) or separately based on whether at least one wild-type *Pappa2* allele was present (Fisher’s Exact Test *P* = 0.33) or not (Fisher’s Exact Test *P* = 1.00) (Table 2). The number of times a female was paired with a male before becoming pregnant did not differ between *Mmp9*^*−/−*^ and other females (F_1,59_ = 0.18; *P* = 0.67), and was not affected by whether at least one wild-type *Pappa2* allele was present (F_1,59_ = 2.22; *P* = 0.14), or by the interaction between these two factors (F_1,59_ = 0.10; *P* = 0.76) (Table 2). Since the number of times a female was paired with a male varied only from 1 to 6, we also analysed these data using a non-parametric Wilcoxon test. Again there was no difference between *Mmp9*^*−/−*^ and other females (*P* = 0.71), pooling matings with and without at least one wild-type *Pappa2* allele present.

**Table 2:** Effects of *Mmp9* and *Pappa2* deletion on fertility including F2 and BC females, i.e., females collected at G16 or G18

	No wild-type *Pappa2* alleles *Mmp9* mating type		At least one wild-type *Pappa2* allele *Mmp9* mating type	
	0–1	2–4	0–1	2–4
# matings for pregnancy^a^	2.3 ± 0.4	2.3 ± 0.3	2.7 ± 0.3	2.9 ± 0.3
# females that became pregnant	11	16	19	17
# females that did not become pregnant	2	9	7	6
# females with no failed matings^b^	9	18	18	19
# females with failed mating^b^	4	7	8	4

^a^Least-squares means ± standard error from a general linear model including *Mmp9* mating type, whether mating had any wild-type *Pappa2* alleles, and the interaction between these two terms

^b^A failed mating was defined as when a vaginal plug was detected following mating, but pregnancy did not develop; this analysis includes females that subsequently became pregnant, and those that never became pregnant

**Fig. 1:**
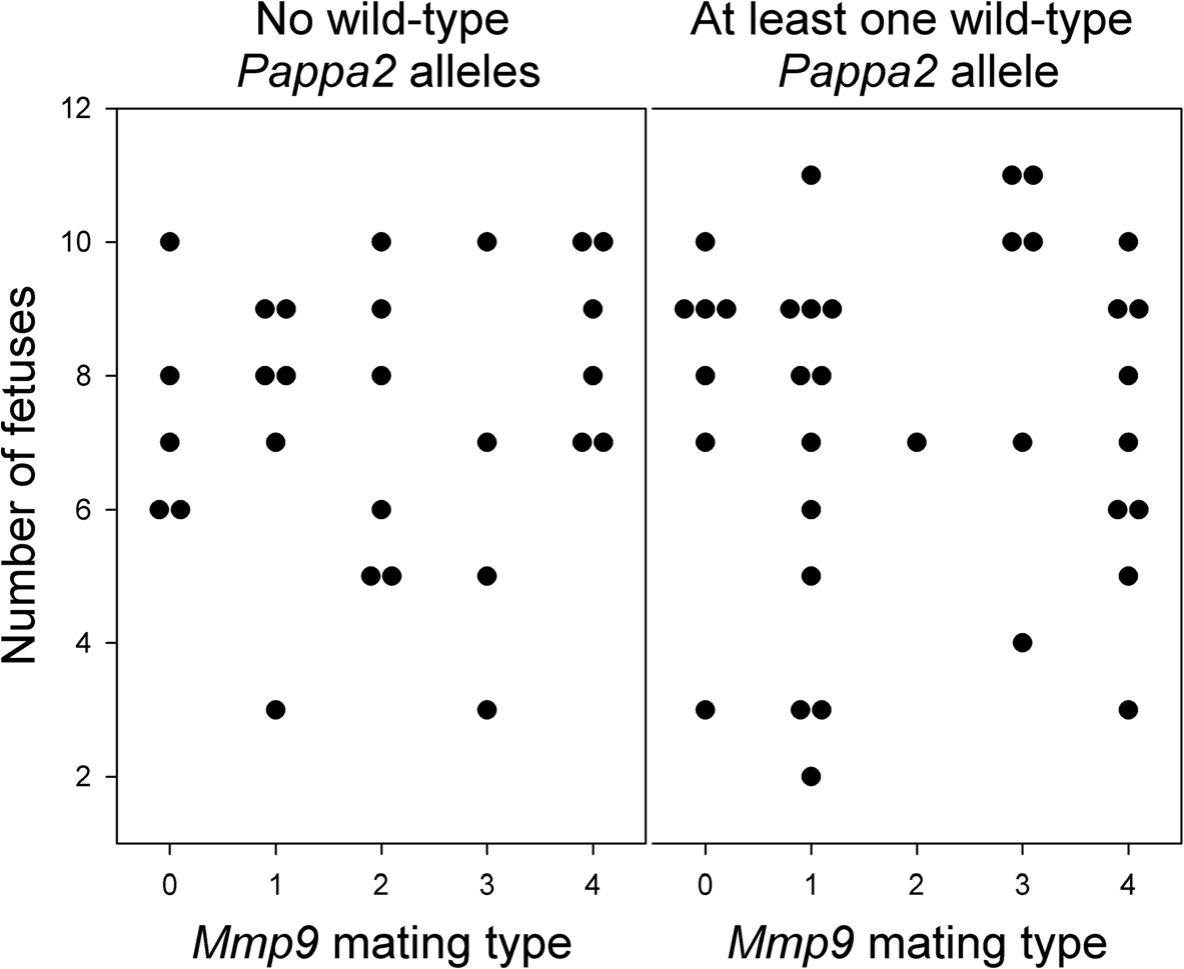
Effect of *Mmp9* and *Pappa2* deletion on the number of fetuses, including females collected at either G16 or G18

We also found no evidence of reduced fecundity or increased fetal loss in *Mmp9*^*−/−*^ females. Including females collected at either G16 or G18, the number of fetuses did not differ between *Mmp9*^*−/−*^ and other females (F_1,58_ = 0.28; *P* = 0.60), and was not affected by whether at least one wild-type *Pappa2* allele was present (F_1,58_ = 0.00; *P* = 0.99) or the interaction between these two factors (F_1,58_ = 0.17; *P* = 0.68) (Fig. 1). The number of putative embryo resorptions ranged from 0 to 4, and tended to be slightly lower in *Mmp9*^*−/−*^ females (mean = 0.6) than in other females (mean = 0.9; Wilcoxon test *P* = 0.25). The proportion of females that had at least one resorption did not differ between *Mmp9* genotypes (11/30 *Mmp9*^*−/−*^ females vs. 16/32 other females; Fisher’s Exact Test *P* = 0.32).

**Fig. 2:**
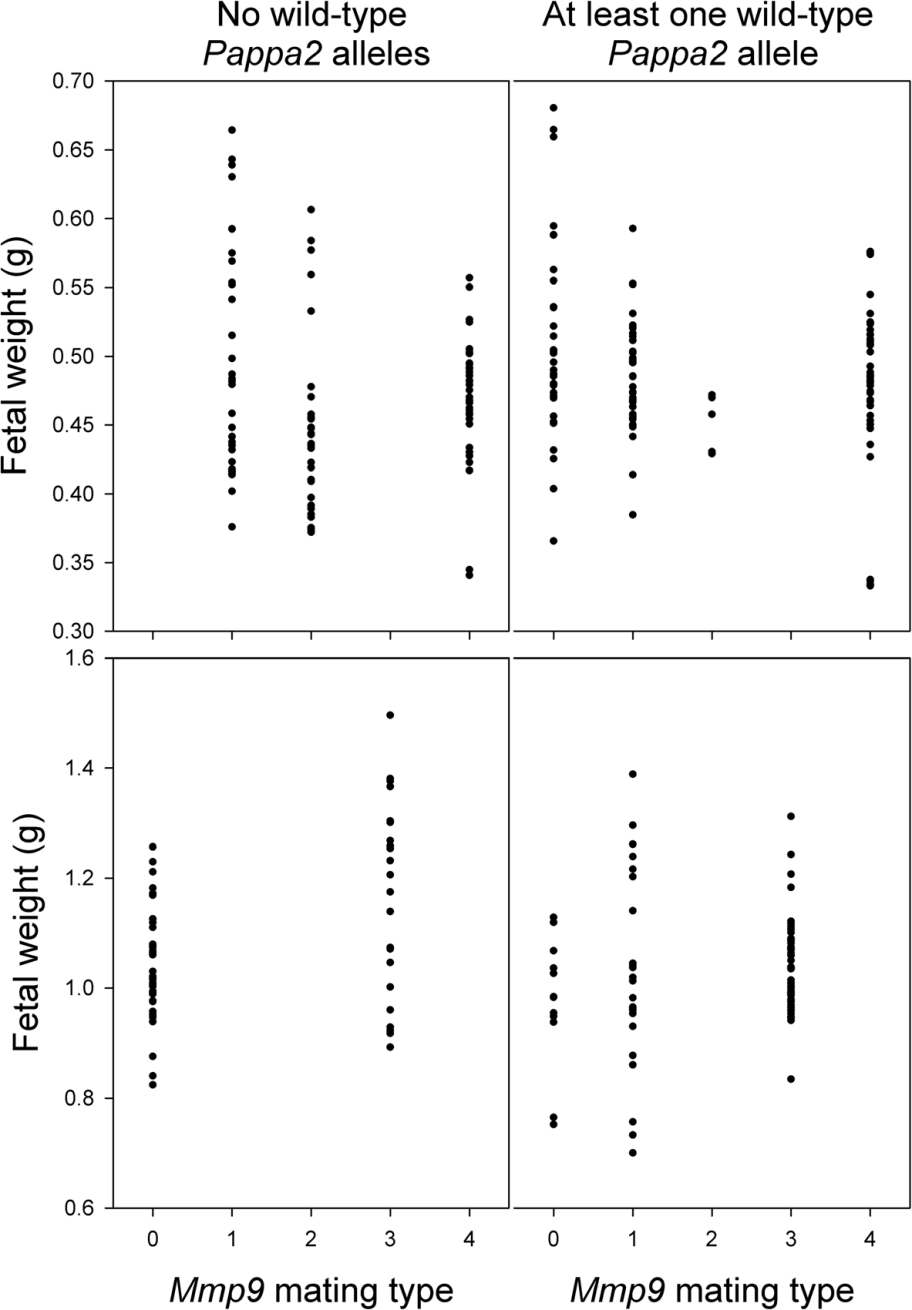
Effect of *Mmp9* and *Pappa2* deletion on fetal weight at G16 (upper panels) and G18 (lower panels)

**Fig. 3:**
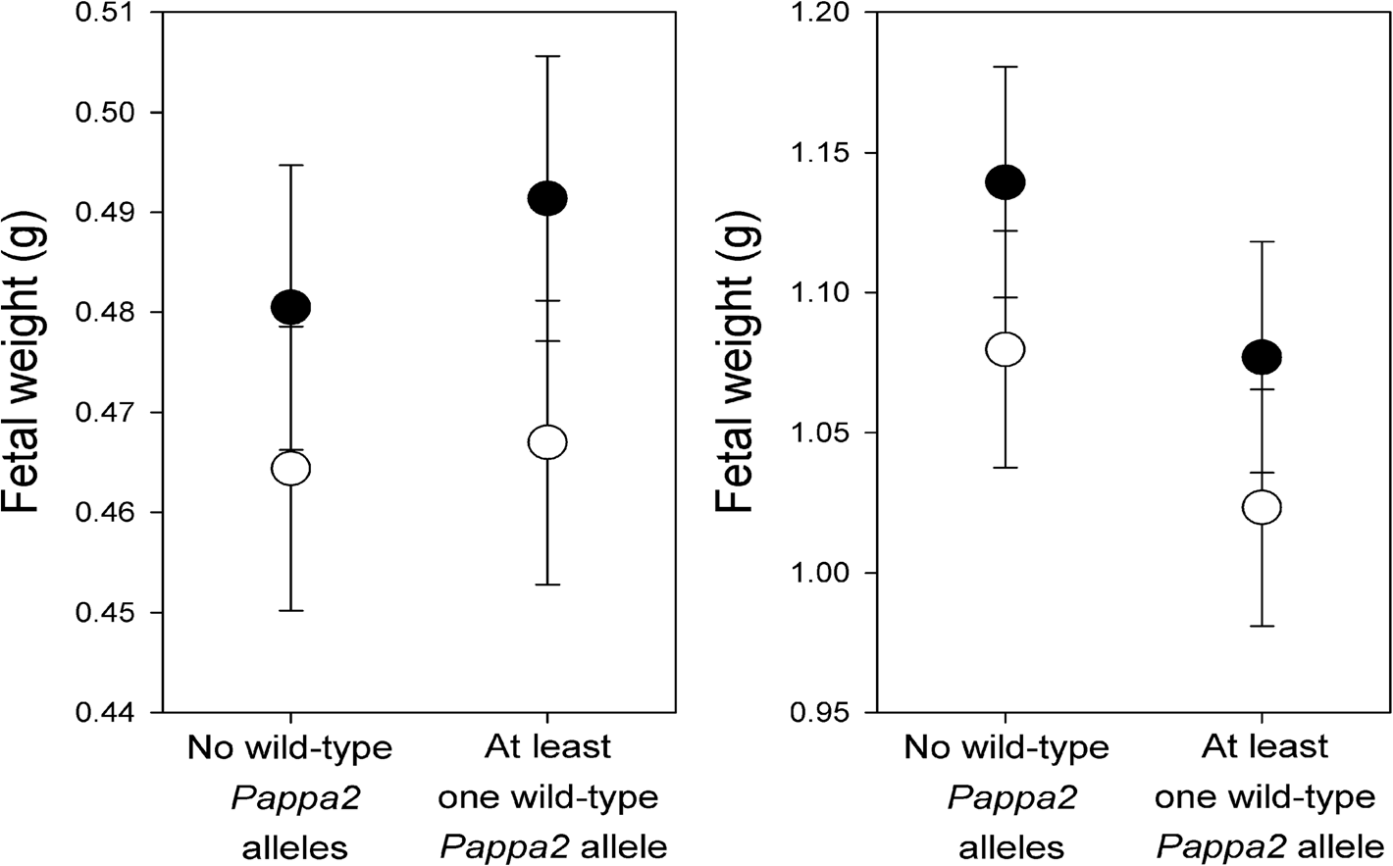
Effect of *Mmp9* and *Pappa2* deletion on fetal weight at G16 (left) and G18 (right) in pregnancies segregating at *Mmp9*. Open symbols are *Mmp9*^*−/−*^ fetuses and solid symbols are their *Mmp9*^*+/−*^ and *Mmp9*^*+/+*^ siblings. Error bars are pooled per *Mmp9* genotype (i.e., pooling *Pappa2* genotype)

At G16, the average fetal mass and average placental mass did not differ between *Mmp9*^*−/−*^ and other females, and was not affected by whether at least one wild-type *Pappa2* allele was present or the interaction between these two factors (controlling for the number of fetuses, which was negatively related to fetal and placental mass; Table 3). There was no excess of very small or runted pups in *Mmp9*^*−/−*^ pregnancies (Fig. 2). There was a tendency for *Mmp9*^*−/−*^ females to have heavier placentae, but this was marginally non-significant (*P* = 0.07, Table 3). Considering only pregnancies with both *Mmp9*^*+/−*^ and *Mmp9*^*−/−*^ fetuses, *Mmp9*^*−/−*^ fetuses and their placentae were significantly lighter than their *Mmp9*^*+/−*^ siblings, but there was no effect of whether a wild-type *Pappa2* allele was present in the pregnancy, and no interaction between *Mmp9* and *Pappa2* (Table 4; Fig. 3). Male fetuses were heavier than female fetuses and had heavier placentae. The number of *Mmp9*^*−/−*^ to *Mmp9*^*+/−*^ conceptuses did not differ from the expected 1:1 ratio (76 *Mmp9*^*−/−*^ vs. 73 *Mmp9*^*+/−*^; χ^2^_1_ = 0.06; *P* = 0.81).

**Table 3:** Effects of *Mmp9* and *Pappa2* deletion on average fetal mass and average placental mass

	Term in model							
	*Mmp9*		*Pappa2*		Mmp9*Pappa2 interaction		Number of fetuses	
G16								
	F_1,36_	P	F_1,36_	P	F_1,36_	P	F_1,36_	P
Average fetal mass	0.89	0.35	0.44	0.51	0.04	0.84	6.51	0.02
Average placental mass	3.50	0.07	0.82	0.37	0.01	0.94	10.76	0.002
G18								
	F_1,15_	P	F_1,15_	P	F_1,15_	P	F_1,15_	P
Average fetal mass	3.41	0.08	1.13	0.31	0.03	0.86	2.08	0.17
Average placental mass	0.61	0.45	1.00	0.33	0.00	0.98	1.11	0.31

Statistics are from general linear models including effects of *Mmp9* deletion (mating types 0 and 1 compared with others), *Pappa2* deletion (whether the cross included at least one functional *Pappa2* allele or not), the interaction between these two factors, and the number of fetuses as a covariate

**Table 4:** Effects of *Mmp9* and *Pappa2* deletion on fetal mass and placental mass in pregnancies segregating for *Mmp9*

	Term in model									
	*Mmp9*		*Pappa2*		Mmp9*Pappa2 interaction		Number of fetuses		Sex of fetus	
G16										
	F_1,16_	P	F_1,18_	P	F_1,16_	P	F_1,18_	P	F_1,15_	P
Fetal mass	9.69	0.007	0.07	0.80	0.40	0.54	2.97	0.10	14.72	0.002
Placental mass	12.63	0.003	0.06	0.80	0.01	0.91	5.12	0.04	31.08	0.0001
G18										
	F_1,10_	P	F_1,11_	P	F_1,10_	P	F_1,11_	P	F_1,11_	P
Fetal mass	9.53	0.012	0.59	0.46	0.03	0.88	7.24	0.02	1.06	0.32
Placental mass	0.81	0.39	1.13	0.31	1.26	0.29	4.49	0.06	17.82	0.0014

These analyses included multiple conceptuses per dam, and so statistics are from repeated measures analyses (with dam as a random factor), including effects of *Mmp9* genotype of conceptus (*Mmp9*^*−/−*^ vs. *Mmp9*^*+/−*^ and *Mmp9*^*+/+*^), *Pappa2* deletion (whether the cross included at least one functional *Pappa2* allele or not), the interaction between these two factors, fetal sex, and the number of fetuses as a covariate

We also studied fetuses at G18, in case growth restriction was more apparent later in pregnancy. As at G16, the average fetal mass and average placental mass did not differ between *Mmp9*^*−/−*^ and other females, and was not affected by whether at least one wild-type *Pappa2* allele was present or the interaction between these two factors, controlling for the number of fetuses (Table 3; Fig. 2). Average fetal mass tended to be lighter in *Mmp9*^*−/−*^ females but this was marginally non-significant (*P* = 0.08, Table 3). Considering only pregnancies segregating at *Mmp9*, *Mmp9*^*−/−*^ fetuses were lighter than their *Mmp9*^*+/−*^ and *Mmp9*^*+/+*^ siblings, but there was no effect of whether a wild-type *Pappa2* allele was present in the pregnancy, and no interaction between *Mmp9* and *Pappa2* (Table 4; Fig. 3). There was no effect of *Mmp9* genotype on placental weight. As at G16, male fetuses had heavier placentae than female fetuses. The genotype ratios did not differ from the expected Mendelian ratios in litters segregating for 2 genotypes (16 *Mmp9*^*−/−*^: 9 *Mmp9*^*+/−*^; χ^2^_1_ = 1.96; *P* = 0.16) or three genotypes (26 *Mmp9*^*−/−*^: 40 *Mmp9*^*+/−*^: 12 *Mmp9*^*+/+*^; χ^2^_1_ = 5.08; P = 0.08). Though non-significant, the trend was for an excess of *Mmp9*^*−/−*^ conceptuses.

**Fig. 4:**
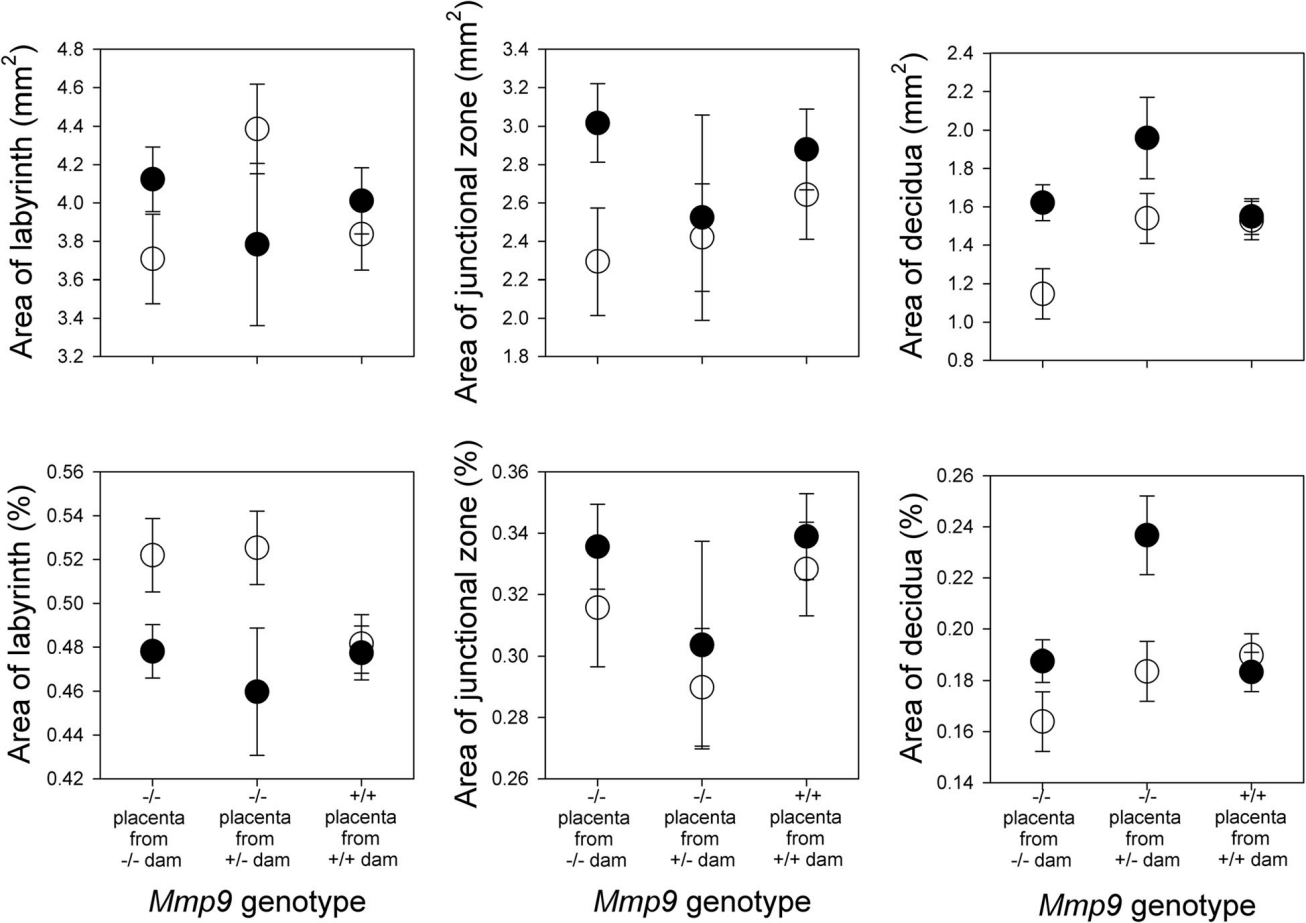
Effect of *Mmp9* and *Pappa2* deletion on the areas of the labyrinth, junctional zone and decidua in absolute terms (upper panels) and as a percentage of the total area (lower panels). Open symbols are pregnancies without a functional *Pappa2* allele and solid symbols are pregnancies with at least one functional *Pappa2* allele. Error bars are from repeated measures analyses (with dam as a random factor), including effects of *Mmp9* group, *Pappa2* deletion, the interaction between these two factors, and fetal sex

**Fig. 5:**
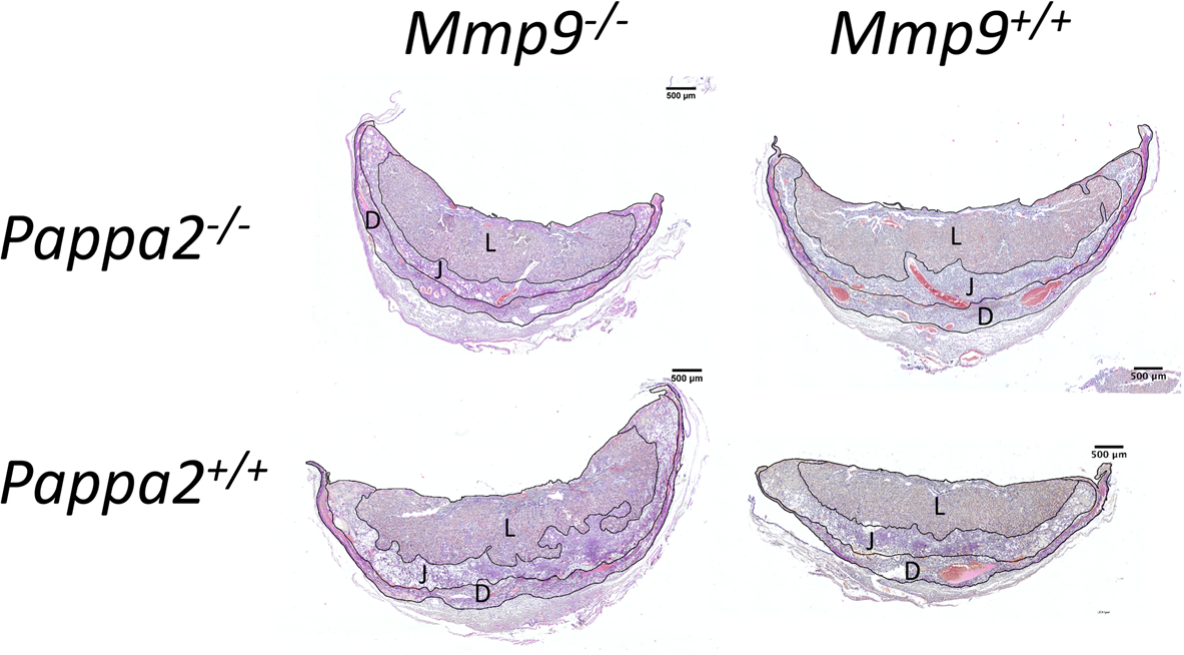
Representative images of G16 placental sections from *Mmp9*^*−/−*^ placentae from *Mmp9*^*−/−*^ dams and *Mmp9*^*+/+*^ placentae from *Mmp9*^*+/+*^ dams, with and without *Pappa2*, showing the outlined labyrinth (L), junctional zone (J) and decidua (D). All placentae are from female fetuses

At G16, we observed little effect of *Mmp9* deficiency on the areas of the labyrinth, junctional zone or decidua, either in absolute terms or in terms of each component as a percentage of the total area (Table 5; Figs. 4 and 5). There was generally no interaction between *Mmp9* deficiency and whether at least one wild-type *Pappa2* allele was present (Table 5; Fig. 4), although *Mmp9*^*−/−*^ placentae from *Mmp9*^*−/−*^ dams with no *Pappa2* had slightly smaller decidua area, when measured as a percentage of the total area (Table 5; Fig. 4). Pregnancies with no *Pappa2* had smaller deciduas in absolute terms, and had larger labyrinths and smaller deciduas as a percentage of the total area, compared to pregnancies where at least one wild-type *Pappa2* allele was present (Table 5; Fig. 4). Placentae of male fetuses had larger junctional zone areas in absolute terms and, as a percentage of the total area, had larger junctional zones and smaller labyrinths.

**Table 5:** Effects of *Mmp9* and *Pappa2* deletion on the areas of the labyrinth, junctional zone and decidua, either in absolute terms, or as a percentage of total area

	Term in model							
	*Mmp9* ^a^		*Pappa2*		Mmp9*Pappa2 interaction		Sex of fetus	
	F_2,28_	P	F_1,28_	P	F_2,28_	P	F_1,20_	P
Absolute								
Labyrinth	0.20	0.82	0.00	0.98	1.64	0.21	0.10	0.75
Junctional Zone	0.38	0.69	1.93	0.18	0.69	0.51	14.76	0.001^b^
Decidua	3.17	0.06	7.89	0.01	2.62	0.09	2.90	0.10
Percentage of total								
Labyrinth	1.15	0.33	6.89	0.01	1.80	0.18	15.01	0.001^b^
Junctional Zone	1.41	0.26	0.79	0.38	0.04	0.96	11.63	0.003^b^
Decidua	4.10	0.03	6.97	0.01	3.96	0.03	0.00	0.98

These analyses included multiple conceptuses per dam, and so statistics are from repeated measures analyses (with dam as a random factor), including effects of *Mmp9* group, *Pappa2* deletion (whether the cross included at least one functional *Pappa2* allele or not), the interaction between these two factors, and fetal sex

^a^In these analyses, there were three *Mmp9* groups: *Mmp9*^*−/−*^ placentae from *Mmp9*^*−/−*^ dams, *Mmp9*^*−/−*^ placentae from *Mmp9*^*+/−*^ dams, and *Mmp9*^*+/+*^ placentae from *Mmp9*^*+/+*^ dams

^b^Area of the junctional zone, both absolute and as a percentage of the total area, was larger in males than females, while the area of the labyrinth as a percentage of the total was smaller in males

**Fig. 6:**
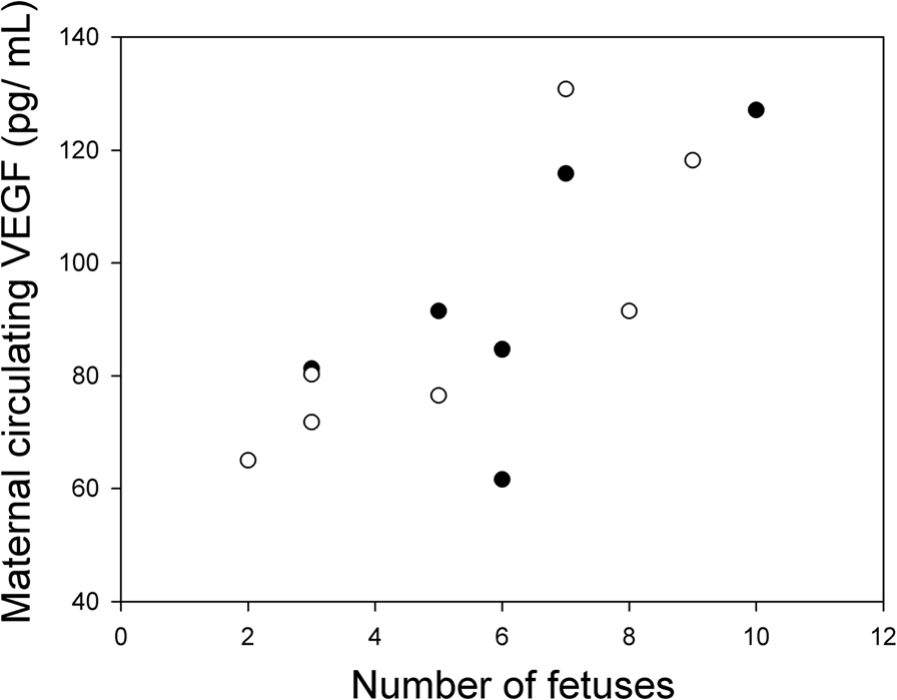
Relationship between number of fetuses and VEGF levels in the maternal circulation of *Mmp9*^*−/−*^ dams (open symbols) and *Mmp9*^*+/+*^ dams (solid symbols) at G16. All pregnancies have at least one functional copy of *Pappa2*

The previous report of *Mmp9* deletion mice [24] described reduced serum VEGF levels in *Mmp9*^*−/−*^ females. We analysed VEGF levels at G16 in a subset of pregnancies of *Mmp9*^*−/−*^ and *Mmp9*^*+/+*^ females, all with at least one wild-type *Pappa2* allele. VEGF levels did not differ between *Mmp9*^*−/−*^ and *Mmp9*^*+/+*^ females (F_1,10_ = 0.12; *P* = 0.73), but were positively related with the number of conceptuses (F_1,10_ = 13.70; *P* = 0.004; Fig. 6).

## Discussion

In humans, placental PAPP-A2 is upregulated in preeclampsia [3–7] and fetal growth restriction [8]. However, deletion of *Pappa2* in mice has little effect on pregnancy outcome [16, 21], suggesting that the upregulation of PAPP-A2 in human pregnancy complications may represent a compensatory response [10, 22, 23]. If PAPP-A2 is important in compensating for placental insufficiency, it would be expected that its absence would exacerbate the effects of placental dysfunction. Previously, *Mmp9* deficiency has been reported to cause placental abnormalities resulting in growth restriction [24]. We observed that *Mmp9*^*−/−*^ fetuses were lighter than *Mmp9*^*+/−*^ siblings, but this difference was not exacerbated by deletion of *Pappa2*. Therefore, our results do not provide evidence that PAPP-A2 contributes to placental mechanisms that compensate for poor fetal growth.

Surprisingly, we found no effect of *Mmp9* deletion, with or without deletion of *Pappa2*, on fertility, fecundity, pregnancy loss, fetal loss or placental structure. While the publication describing *Mmp9* deletion as a model of preeclampsia and intrauterine growth restriction reported “as much as a 50% reduction in litter size” [24], no data were presented, and the original report of *Mmp9* deletion described a much more modest reduction in litter size (1.6 pups) [27]. In our experiments, *Mmp9*^*−/−*^ females were compared with control siblings, and no experimental females were daughters of *Mmp9*^*−/−*^ females. It is therefore possible that by avoiding maternal effects of *Mmp9* deletion, the severity of the deletion was reduced; whether previous reports [24, 27] used our breeding scheme is not clear.

The previous report of the effects of *Mmp9* deficiency on pregnancy also reported reduced levels of VEGF in the maternal circulation [24]. VEGF influences placental angiogenesis, and maternal circulating VEGF levels are reduced in preeclamptic human pregnancies [28]. While there was no effect of *Mmp9* deficiency on circulating maternal VEGF in the present study, we found a positive association between VEGF levels and the number of conceptuses. The previous report of reduced VEGF in *Mmp9*^*−/−*^ females [24] may therefore have been due to reduced numbers of conceptuses, rather than to placental pathology.

In addition to some modest effects of *Mmp9* and *Pappa2* deletion, we observed more robust differences between the sexes in fetal mass, placental mass, and placental structure at G16, as well as placental mass at G18. These differences may reflect sex-specific strategies for fetal growth and placental function [29] with potential long term effects on offspring health [30].

## Conclusions

Previous work reported that deletion of *Mmp9* reduced pregnancy rates and implantation success following embryo transfer, decreased litter size, and increased rates of fetal demise and growth restriction [24]. In contrast, we found only modest effects of *Mmp9* deletion on fetal growth, and found no effects on the number of fetuses, the number of putative embryo resorptions, or female fertility (number of matings required to achieve pregnancy, the proportion of females that became pregnant, or the number of females with failed matings). The difference in results between our study and previous work may have been due to our experimental design, which compared *Mmp9*^*−/−*^ females with control siblings and thus avoided confounding maternal effects. The effects of *Mmp9* deficiency were not exacerbated by the deletion of *Pappa2*, and therefore our results do not support the hypothesis that PAPP-A2 upregulation in human pregnancy complications represents a compensatory response to ameliorate placental growth and development. Our results provide insight into the role of PAPP-A2 dysregulation in devastating pregnancy complications, which may inform the use of this protein as an early biomarker of placental health [9, 10]. Our work also serves as a caution regarding the use of *Mmp9* deficiency as a model of these complications.

## Additional files

Additional file 1: Genotype ratios and postnatal growth of F2 and BC populations. (DOCX 326 kb)

Additional file 2: Datasets analysed in this study. (XLSX 69 kb)

## Abbreviations

BC: Backcross
F1: First generation
F2: Second generation
G16, G18: day 16 or 18 of gestation, where the day after mating = day 0
IGF: Insulin-like growth factor
IGFBP: Insulin-like growth factor binding protein
MMP9: Matrix metalloproteinase-9
PAPP-A2: Pregnancy-associated plasma protein-A2
VEGF: Vascular endothelial growth factor
