# Supplementary material for: PAPP-A2 deficiency does not exacerbate the phenotype of a mouse model of intrauterine growth restriction
Source: Reprod Biol Endocrinol. 2018 Jun 12;16:58. doi: 10.1186/s12958-018-0376-4 (PMC5996520; doi:10.1186/s12958-018-0376-4)
Supplement: Supplementary file 1 — Genotype ratios and postnatal growth of F2 and BC populations. (DOCX 326 kb) [file 12958_2018_376_MOESM1_ESM.docx]

**Supplementary results**

*Postnatal growth*

Postnatal growth was analysed using general linear models (proc GLM) including terms for dam, sex, *Mmp9* genotype, *Pappa2* genotype, and the *Mmp9*Pappa2* interaction. The F2 population was weaned at three weeks of age, at which time *Mmp9^-/-^* pups were lighter than their siblings (F_2,130_ = 8.57, P = 0.0003), as were *Pappa2^-/-^* pups (F_2,130_ = 14.99, P < 0.0001), but the interaction between *Mmp9* and *Pappa2* genotype was marginally non-significant (F_4,130_ = 2.10, P = 0.084; Figure S1). Males were heavier than females (F_1,130_ = 10.87, P = 0.0013). At 6 weeks of age, *Mmp9^-/-^* mice were again lighter than their siblings (F_2,128_ = 9.90, P = 0.0001), as were *Pappa2^-/-^* mice (F_2,128_ = 29.98, P < 0.0001). There was also significant interaction between *Mmp9* and *Pappa2* genotype (F_4,128_ = 3.78, P < 0.006), whereby the differences among *Mmp9* genotypes were greatest among *Pappa2^+/+^* mice (Figure S1). Males were heavier than females (F_1,128_ = 491.10, P < 0.0001). At 8 weeks of age, *Mmp9^-/-^* and *Pappa2^-/-^* mice remained lighter (*Mmp9*: F_2,106_ = 7.75, P = 0.0007; *Pappa2*: F_2,106_ = 23.95, P < 0.0001) but there was no longer an interaction between *Mmp9* and *Pappa2* genotype (F_4,106_ = 1.28, P = 0.28; Figure S1). Males were heavier than females (F_1,106_ = 438.96, P < 0.0001). The BC population was weaned later than the F2 population, at around 4 weeks of age. *Pappa2^-/-^* pups were lighter than their siblings (F_1,57_ = 16.39, P = 0.0002), and males were heavier than females (F_1,57_ = 15.45, P = 0.0002), but there was no effect of *Mmp9* genotype (F_1,57_ = 2.77, P = 0.10) or the interaction between *Mmp9* and *Pappa2* genotype (F_1,57_ = 2.32, P = 0.13; Figure S2). The BC population was not weighed between weaning and the time the females were paired.

Analysing the F2 and BC together, *Mmp9^-/-^* females were lighter immediately prior to conception (F_2,53_ = 3.56, P = 0.04), as were *Pappa2^-/-^* females (F_2,53_ = 32.62, P < 0.0001), but there was no interaction between *Mmp9* and *Pappa2* genotype (F_2,53_ = 0.67, P = 0.62; Figure S3).

*Genotype ratios*

In the F2 population, the frequency of *Mmp9* genotypes deviated from the expected 1:2:1 ratio (χ^2^_2_ = 8.8, P = 0.012), with an excess of *Mmp9^-/-^* and a deficiency of *Mmp9^+/-^* individuals (Table S1). *Pappa2* genotypes did not deviate from expected 1:2:1 ratio (χ^2^_2_ = 3.9, P = 0.14). In the BC population, the frequency of *Mmp9* genotypes did not deviate from the expected 1:1 ratio (χ^2^_2_ = 2.4, P = 0.12; Table S2), although there was a trend towards an excess of *Mmp9^-/-^*. *Pappa2* genotypes did not deviate from expected 1:1 ratio (χ^2^_2_ = 0.13, P = 0.72). A potential explanation for the excess of *Mmp9^-/-^* genotypes is that some *Mmp9^+/-^* mice were erroneously genotyped as *Mmp9^-/-^*. However, genotyping of fetuses provided a check of parental genotypes, and only in 1 case out of 40 putative *Mmp9^-/-^* parents did we detect a *Mmp9^+/-^* parent that had been erroneously genotyped as *Mmp9^-/-^*.

Table 1. Genotype frequencies in the F2 population.

|  | *Pappa2^-/-^* | *Pappa2^+/-^* | *Pappa2^+/+^* | Total |
| --- | --- | --- | --- | --- |
| *Mmp9^-/-^* | 23 | 22 | 13 | 58 |
| *Mmp9^+/-^* | 18 | 40 | 11 | 69 |
| *Mmp9^+/+^* | 12 | 16 | 13 | 41 |
| Total | 53 | 78 | 37 | 168 |

Table S2. Genotype frequencies in the BC population.

|  | *Pappa2^-/-^* | *Pappa2^+/-^* | Total |
| --- | --- | --- | --- |
| *Mmp9^-/-^* | 20 | 21 | 41 |
| *Mmp9^+/-^* | 16 | 12 | 28 |
| Total | 36 | 33 | 69 |


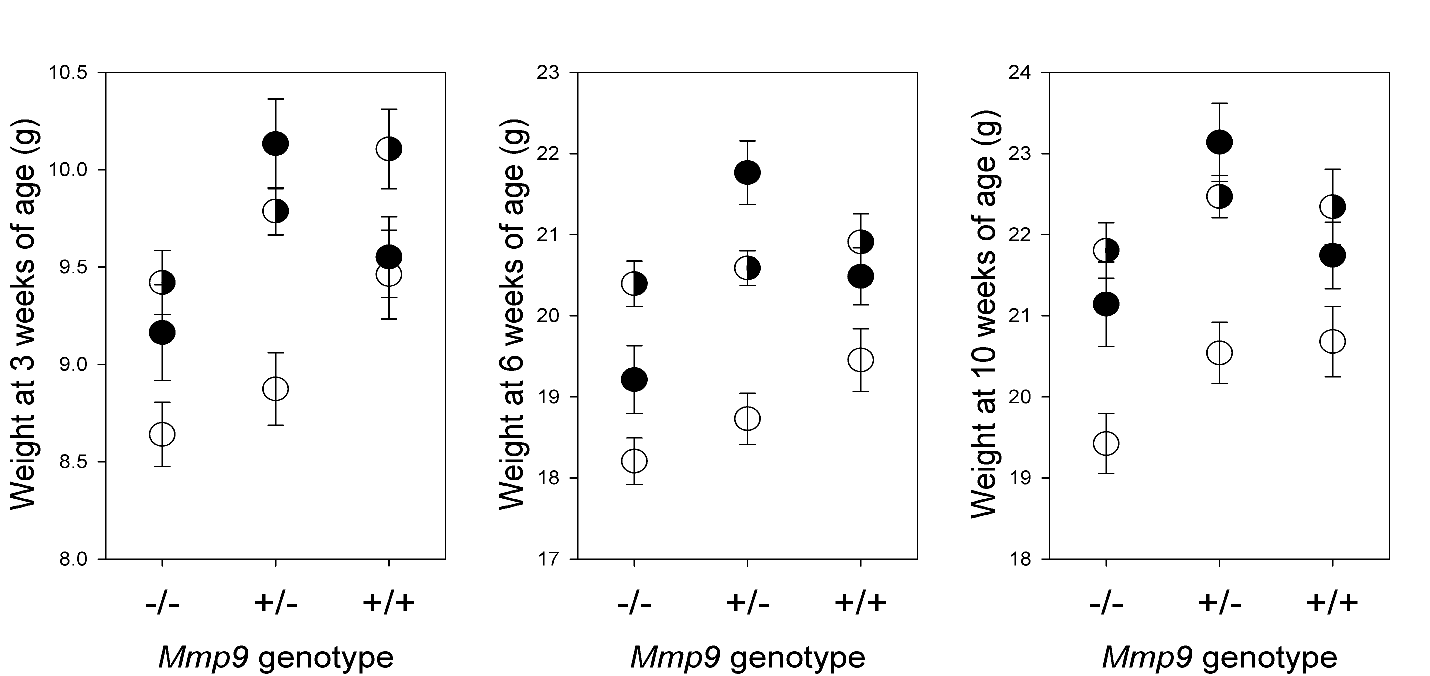


Figure S1. Effects of *Mmp9* and *Pappa2* genotype on body weight at 3, 6 and 8 weeks of age in the F2 population (open symbols: *Pappa2^-/-^* ; half open symbols: *Pappa2^+/-^* ; closed symbols: *Pappa2^+/+^*). Values are least squares means ± standard error from a general linear model including effects of dam, sex, *Mmp9* genotype, *Pappa2* genotype, and the *Mmp9*Pappa2* interaction.


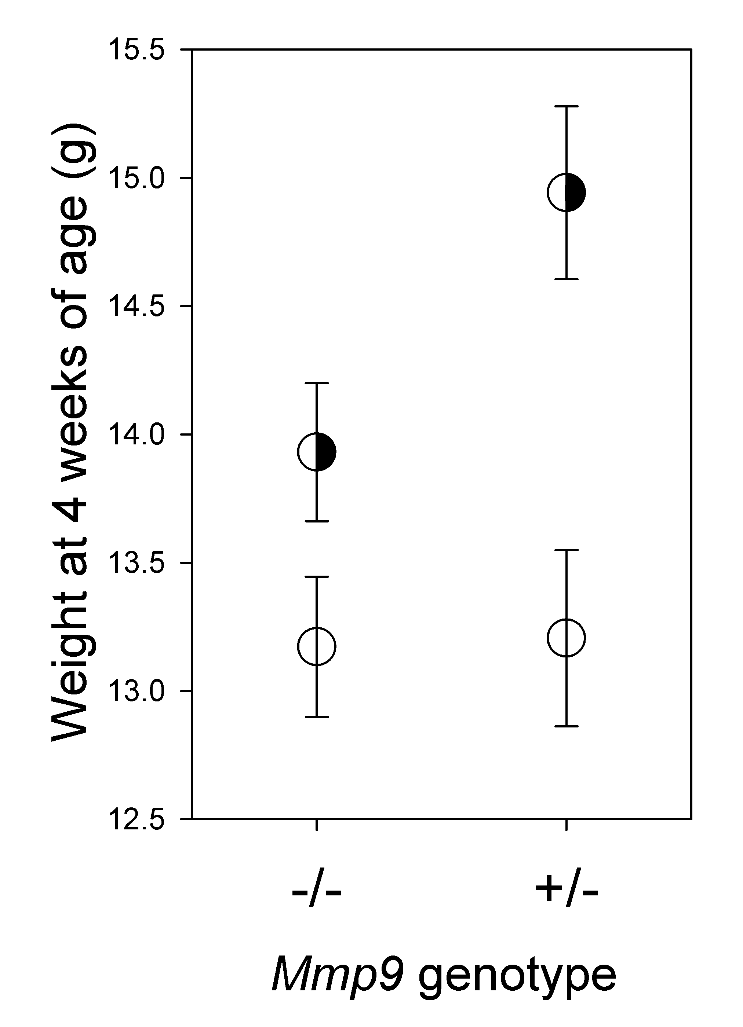


Figure S2. Effects of *Mmp9* and *Pappa2* genotype on body weight at weaning in the BC population (open symbols: *Pappa2^-/-^* ; half open symbols: *Pappa2^+/-^*). Values are least squares means ± standard error from a general linear model including effects of dam, sex, *Mmp9* genotype, *Pappa2* genotype, and the *Mmp9*Pappa2* interaction.


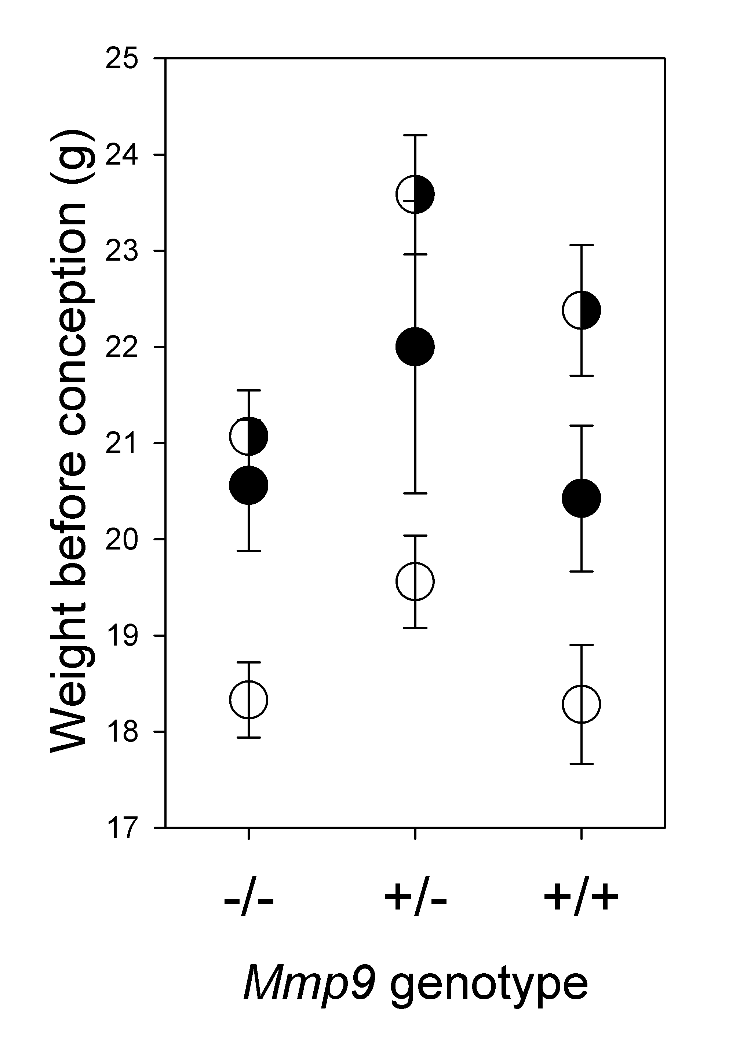


Figure S3. Effects of *Mmp9* and *Pappa2* genotype on body weight of females before conception in the combined F2 and BC population (open symbols: *Pappa2^-/-^* ; half open symbols: *Pappa2^+/-^* ; closed symbols: *Pappa2^+/+^*). Values are least squares means ± standard error from a general linear model including effects of *Mmp9* genotype, *Pappa2* genotype, and the *Mmp9*Pappa2* interaction.
